# The beauty of being (label)-free: sample preparation methods for SWATH-MS and next-generation targeted proteomics

**DOI:** 10.12688/f1000research.2-272.v2

**Published:** 2014-04-07

**Authors:** Jakob Vowinckel, Floriana Capuano, Kate Campbell, Michael J. Deery, Kathryn S. Lilley, Markus Ralser

**Affiliations:** 1 Cambridge Systems Biology Centre and Dept. of Biochemistry, University of Cambridge, Cambridge, CB2 1GA, UK; 2 Division of Physiology and Metabolism, MRC National Institute for Medical Research, London, NW7 1AA, UK

**Keywords:** label-free quantitative proteomics, in gel digest, filter-aided sample preparation, RapiGest, acetonitrile-based protein digestion, SWATH, data dependent acquisition.

## Abstract

The combination of qualitative analysis with label-free quantification has greatly facilitated the throughput and flexibility of novel proteomic techniques. However, such methods rely heavily on robust and reproducible sample preparation procedures. Here, we benchmark a selection of *in gel*, *on filter*, and *in solution* digestion workflows for their application in label-free proteomics. Each procedure was associated with differing advantages and disadvantages. The *in gel *methods interrogated were cost effective, but were limited in throughput and digest efficiency. *Filter-aided* sample preparations facilitated reasonable processing times and yielded a balanced representation of membrane proteins, but led to a high signal variation in quantification experiments. Two *in solution* digest protocols, however, gave optimal performance for label-free proteomics. A protocol based on the detergent *RapiGest* led to the highest number of detected proteins at second-best signal stability, while a protocol based on acetonitrile-digestion, *RapidACN*, scored best in throughput and signal stability but came second in protein identification. In addition, we compared label-free data dependent (DDA) and data independent (SWATH) acquisition on a TripleTOF 5600 instrument. While largely similar in protein detection, SWATH outperformed DDA in quantification, reducing signal variation and markedly increasing the number of precisely quantified peptides.

## Introduction

Mass spectrometry (MS)-based proteomics facilitates the identification of a large number of proteins in a single experiment ^1– 3^. As a result this technique has been established as a powerful complement to the classic tools of protein chemistry, such as western-blotting or enzyme-linked immunosorbent (ELISA) assays, which are of considerably lower throughput and specificity. Whereas traditional proteomic workflows mainly aimed to identify proteins, quantification has meanwhile become a major focus of technological development in this field ^4, 5^. On a quantitative liquid chromatography/mass spectrometry (LC-MS) platform the amount of analyte and the corresponding chromatographic peak area are in linear correlation, hence concentration values are obtained through comparison with reference standards ^6^. A technically powerful approach for protein quantification involves the use of isotope-labelled standards that show a similar structure and chromatographic behaviour to the target molecule, but are distinguishable from the target by mass ^7^. When added at an early stage of the quantification workflow, they allow for correction of analyte loss during sample preparation and analysis, hence rendering the quantification experiment robust. However, the requirement for isotope-labelled standards makes proteomic workflows expensive and reduces flexibility, as their production is laborious and applicable only to samples for which these standards can be obtained or generated (please see Discussion). Moreover, as both the analyte and standard need to be measured, they double the analyte load for the mass spectrometer. Consequently, recent developments that have enabled label-free peptide and protein quantification have attracted much attention ^8– 12^. In a label-free experiment, quantification is achieved through comparison of peak areas obtained for an analyte under two or more biological conditions; for instance to compare a wild-type versus a mutant, a compound-exposed versus a control condition, or a biological time series ^13– 16^. Upon normalisation, ideally to one or more unaffected internal standards, this approach yields a relative expression value for the target protein. This measure is then used to evaluate whether the expression of the target is altered between the conditions tested. In the case of high sequence coverage, absolute quantities may also be estimated, as peak intensities obtained for the best ionizing peptides correlate in approximation with their absolute concentration ^10, 12^.

The absence of an internal standard spiked early in sample preparation protocols means that label-free methods are sensitive to technical variance, consequently, label-free proteomics requires high instrument performance and standardization of sample preparation methods. In terms of instrumentation, limitations arise from the linear range of the mass spectrometer and the sample capacity of the liquid chromatography. Moreover, in untargeted proteomics, the stochastic nature of data-dependent acquisition methods, where ions are selected for analysis based on their intensity, reduces the number of quantifiable peptides to only those fragmented in all samples ^17, 18^. This problem is a consequence of the high number of co-eluting peptides that may considerably exceed the mass spectrometer’s sampling speed when analysing full proteomes, a problem that is amplified by the high number of replicates used in a label-free study. By facilitating data-independent acquisition, where all ions are fragmented irrespective of their intensity, recent studies have demonstrated the possibility of circumventing the need of isolating individual peptides ^11, 17^. One such method, pioneered by the Waters Corporation, is termed MS ^E, 11^. In this approach fragment ions are assumed to have the same elution profiles as their precursors; this similarity is then used to pair fragments and precursors when a number of parent ions are co-fragmented. In the typical workflow, fragment pairs and their corresponding precursor ions are retrospectively paired for database searching ^11^. More recently, in a workflow termed SWATH, a mass range relevant for peptide-based proteomics (400–1200 m/z) is scanned in 25 m/z windows, in which all ions that fall into that window are simultaneously fragmented (MS/MS ^all^). Quantification is then conducted based on the peak areas of extracted ion chromatograms (XIC), which are computationally reconstituted from the merged spectra on the basis of both experimental and *in silico* generated spectral information ^17^.

Sample preparation techniques are equally important for the performance of a label-free experiment, and easier to optimize on a daily basis than the mass spectrometer’s properties. The main objective for a label-free sample preparation method is to obtain stable peak intensities between replicate sample preparations. Consequently, the ideal workflow avoids processing steps that are prone to stochastic analyte losses, and the LC-MS set up is operated in such a way that the instrument's dynamic range does not become exhausted. These objectives may differ to classic shotgun proteomics, where the number of identifiable peptides and proteins is the most important value, and a higher variation in signal intensities is acceptable. For this reason, a sample preparation method and LC-MS/MS configuration, which is ideal for identifying a maximum number of proteins, may be sub-optimal for label-free quantification, and *vice versa*. For instance, pre-fractionation of the sample prior to the LC-MS/MS analysis, a popular strategy to improve peptide identification, adds another level of complexity to the sample preparation, increasing the signal variability and thus, is avoided wherever possible.

The main objective of the study presented here is to benchmark proteomic sample preparation methods for their suitability in label-free proteomic studies. We compare popular sample protocols that are based on *in gel* ^19^, *filter-aided* ^20, 21^ and *in solution* ^9, 22^ digestion procedures. Processing identical proteome samples obtained from budding yeast, and acquiring proteomic data without further prefractionation on two LC-MS/MS platforms, these methods were compared by their performance in sample preparation, their precision in label-free quantification experiments and their effectiveness in terms of time and reagents. Through the analysis of these samples on a 5600 QqTOF ^23^ instrument operating in either a data-dependent mode or SWATH ^24^ mode, this study concludes with an evaluation of data-dependent and data independent acquisition, and suggestions for the optimal protocol selection.

## Experimental section

### Reagents

For sample preparation the following reagents were used: Water ULC-MS grade (Greyhound Cat. No. 23214125), formic acid 99% ULC-MS (Greyhound Cat. No. BIO-06914131) and acetonitrile ULC-MS grade (Greyhound Cat. No. Bio-012041-2.5L). Chemicals were obtained from Sigma, with the exception of RapiGest SF (Waters, Cat. No. 186001861), trypsin (Promega Cat. No. V5111), Lys-C (Promega, Cat. No. No. V1071), complete EDTA-free protease inhibitor cocktail tablets (used in the eFASP protocol) (Roche Cat. No. 11873580001), dithiothreitol (Melford Cat No. MB1015), ammonium bicarbonate (Fluka Cat. No. 40867-50G-F), sodium dodecyl sulfate (Melford Cat. No. S1030), 30% acrylamide/0.8% bis-acrylamide (Protogel, Geneflow Limited Cat. No. EC-890), tri-n-butylphosphate (Fluka Cat. No. 90820-100ML) and BCA Protein assay kit (Pierce Cat. No. 23225).

### Preparation of yeast cells

All experiments were conducted using a single culture derived from a single colony of the yeast strain BY4741 ^25^. The strain was transferred to yeast peptone dextrose (YPD) media prepared as described in ^26^ and incubated at 30°C at 200 rpm overnight (ON). Subsequently the ON culture was diluted to an optical density (OD _600_) of 0.2 as measured on an Ultrospec 2000 (Amersham) spectrophotometer, and incubated at 30°C until reaching OD _600_ = 2. The culture was split into aliquots corresponding to 10 OD _600_ units, and stored at -80°C until processing.

### Protein sample preparation for DDA and SWATH analysis

A detailed protocol for each of the six procedures is available in the Supplementary Materials (found at the end of the document in the offline version) (see Supplementary protocol 1– Supplementary protocol 6). In brief, protein samples were prepared from 30 mg (wet weight) of yeast pellet. For the *in gel* digest protocols, protein extraction was performed either in 200 µl SDT buffer (4% SDS, 100 mM Tris*HCl pH 7.6, 0.1 M dithiothreitol) or 0.05 M ammonium bicarbonate using a Fast-Prep 24 instrument (MP Biomedicals). 50 µg of protein was applied to a denaturing polyacrylamide gel and subjected to electrophoresis (for details please see Supplementary protocol 1 and Supplementary protocol 2). The sample was excised as a single band, cut into pieces, and subjected to tryptic digestion ^27^. For the *filter-aided* protocols (FASP, Supplementary protocol 3 and Supplementary protocol 4) protein extraction was performed either in 200 µl SDT buffer (4% SDS, 100 mM Tris/HCl pH 7.6, 0.1 M dithiothreitol) (FASP, Supplementary protocol 3) or lysis buffer (1% SDS, 10 mM Tris/HCl pH 7.4, 0.15 M NaCl, 1 mM EDTA in PBS) (eFASP, Supplementary protocol 4). For both protocols the digestion was performed directly on filters (Amicon Ultra-0.5 Centrifugal Filter Unit with Ultracel-3 membrane, Millipore). The FASP procedure ( Supplementary protocol 3) involved a treatment with endoproteinase Lys-C (Promega) prior to digestion with trypsin ^20^, while the eFASP protocol ( Supplementary protocol 4) required protein precipitation using tri-n-butylphosphate/acetone/methanol mix (1:12:1) for lipid removal before digestion ^21^. For *in solution* digest protocols ( Supplementary protocol 5 and Supplementary protocol 6) protein extraction was performed either in 200 µl lysis buffer (0.1 M NaOH, 0.05 M EDTA, 2% SDS, 2% β-mercaptoethanol) (RapiGest) or 0.05 M ammonium bicarbonate (RapidACN) ^20^ and using heat or glass-bead lyses on a Fast-Prep 24 instrument (MP Biomedicals), respectively. The *in solution* digest protocol based on the detergent RapiGest included a step of protein precipitation for lipid removal through centrifugation prior to trypsin treatment. For the *in solution* acetonitrile-based digestion protocol, a optional clean-up step using 3 kDa molecular cut off filters (Amicon Ultra-0.5 Centrifugal Filter Unit with Ultracel-3 membrane, Millipore) was performed immediately after trypsin digestion ^9^. Before analysis, samples for DDA or SWATH analysis were supplemented with iRT or HRM (Biognosys) standard peptides, respectively, designed to normalize retention time variations. In order to maximize the proteome depth for the generation of a SWATH ion library, tryptic digests prepared with the RapidACN protocol were separated by high pH reverse phase chromatography before LC-MS/MS analysis. A reverse phase column (Waters, BEH C18, 2.1 × 150 mm, 1.7 µm) was utilised in combination with a 20 mM ammonium formate to 20 mM ammonium formate/80% ACN gradient. Twenty fractions were collected and analysed.

### LC-MS/MS analysis

LC-MS/MS analysis of digested *S. cerevisiae* lysates was performed on a Tandem Quadrupole Time-of-Flight mass spectrometer (AB/Sciex TripleTOF5600) coupled to a Nanospray III Ion Source (AB/Sciex) and nano-HPLC (Eksigent Ultra 2D) (referred to as the TripleTOF platform), or hybrid quadrupole orbitrap mass spectrometer (QExactive, Thermo Scientific) coupled to a Dionex Ultimate 3000 and an Easy-spray nanospray ion source (referred to as QExactive platform).

On the TripleTOF platform, peptide separation was carried out by first removing impurities on a pre-column (C18 PepMap100 column NAN75-15-03-C18-PM, Thermo Fisher Scientific Cat. No. 160321) running isocratically at 100% solvent A at a flow rate of 5 μL min ^-1^ for 6 min. Peptides were then eluted onto the analytical column (Zorbax 300SB-C18 column, 75 µm id × 15 cm 3.5 µm, Agilent Technologies Cat. No. 5065-9911), and separated on a linear gradient of 5–35% solvent B for 155 min at a flow rate of 300 nL min ^-1^. Peptides were injected into the mass spectrometer using 10 µm SilicaTip electrospray emitters (New Objective Cat. No. FS360-20-10-N-20-C12), and the ion source was operated with the following parameters: ISVF = 2500; GS1 = 12; CUR = 25. The data acquisition mode in the DDA experiments was set to obtain a high resolution TOF-MS scan over a mass range 400–1250 m/z, followed by MS/MS scans of 20 ion candidates per cycle with dynamic background subtraction, operating the instrument in high sensitivity mode. The selection criteria for the parent ions included the intensity, where ions had to be greater than 150 cps, with a charge state between 2 and 4. The dynamic exclusion duration was set for 15 s. Collision-induced dissociation was triggered by rolling collision energy ( Supplementary Table 1). The ion accumulation time was set to 250 ms (MS) and to 100 ms (MS/MS). For SWATH MS-based experiments the instrument was tuned to specifically allow a quadrupole resolution of 25 Da/mass selection. An isolation width of 25 Da was set in a looped mode over the full mass range (400–1250 m/z) scan and 32 overlapping windows were constructed ^28^. An accumulation time of 100 ms was set for each fragment ion resulting in a total duty cycle of 3.3 s.

For LC-MS/MS analysis using the QExactive platform, separation of peptides was performed at a flow rate of 300 nL min ^-1^ using a reverse-phase nano column (Easy-spray, Thermo Scientific PepMap C18, 2 µm particle size, 100 Å pore size, 75 µm i.d. × 50 cm length). Peptides were loaded onto a pre-column (Thermo Scientific PepMap 100 C18, 5 µm particle size, 100 Å pore size, 300 µm i.d. × 5 mm length) from the Ultimate 3000 autosampler (Dionex) with 0.1% formic acid for 3 minutes at a flow rate of 10 µL min ^-1^. Polar impurities were removed by running the system isocratically at 100% A at a flow rate of 5 μl min ^-1^ for 6 min. Finally, tryptic peptides were loaded onto the analytical column and separated using a linear acetonitrile gradient of 5–35% B for 155 min at a flow rate of 300 nL min ^-1^. The LC eluant was injected into the mass spectrometer by means of an Easy-spray source (Thermo Fisher Scientific). All m/z values of eluting ions were measured in an Orbitrap mass analyzer, set at a resolution of 70,000. Data dependent scans were employed to automatically isolate the 20 most abundant ions and generate fragment ions by higher energy collisional dissociation (HCD) in the quadrupole mass analyser. Only peptide ions with charge states of 2 ^+^ and above were selected for fragmentation. Finally, the measurement of the resulting fragment ions was performed in the Orbitrap analyser, set at a resolution of 17,500.

### Data processing

Data acquired in DDA mode was analysed by means of either the Paragon ^29^ (ProteinPilot software, AB/Sciex, v. 4.5.0.0, 1654) or the Mascot search algorithm (Matrix Science, version 2.3.02) using the *S. cerevisiae* S288C translated ORF database (based on SGD genome version R64-1-1 ^30^). 156 common contaminant ions (AB/Sciex) were excluded from subsequent analysis. For Paragon searches, the following settings were used: Sample type: Identification; Cys Alkylation: Iodoacetamide; Digestion: Trypsin; Instrument: TripleTOF5600; Special Factors: none; Species: *S. cerevisiae*; Search effort: Thorough ID; Results Quality: 0.05. Only peptides with a confidence score of > 0.05 were considered for further analysis. For Mascot searches, the data was pre-processed using PeakView (AB/Sciex, v. 1.2.0.3) or Proteome Discoverer (Thermo Scientific, v. 1.3) setting carbamidomethylation of cysteine (C) as a fixed modification, oxidation of methionine (M) as a variable modification and allowing a maximum of 2 missed cleavages. Fragment mass tolerance was set to 0.8 Da, and Instrument type was ESI-TRAP. Peptides under a significance threshold of 0.05 were regarded as acceptable.

For the extraction of data acquired in SWATH mode, an ion library for yeast was generated from data acquired in data dependent mode. Spectral data were acquired in DDA mode and analysed using the Paragon search strategy as described above. Detected peptides were then corrected for retention time shifts, and the corresponding spectra were combined leading to a library containing 2800 unique yeast proteins. For extraction of SWATH data and peptide quantification Spectronaut 3 (Biognosys) and Skyline ^31^ were used. In parallel, Skyline was also used for quantification of peptides from data dependent acquisition experiments. Subsequent data analysis was performed with R, ggplot2 package and custom-built scripts. GO analysis was based on the SGD Gene Ontology Slim Mapper.

## Results

### Protocol selection and overall assessment

**Figure 1.**
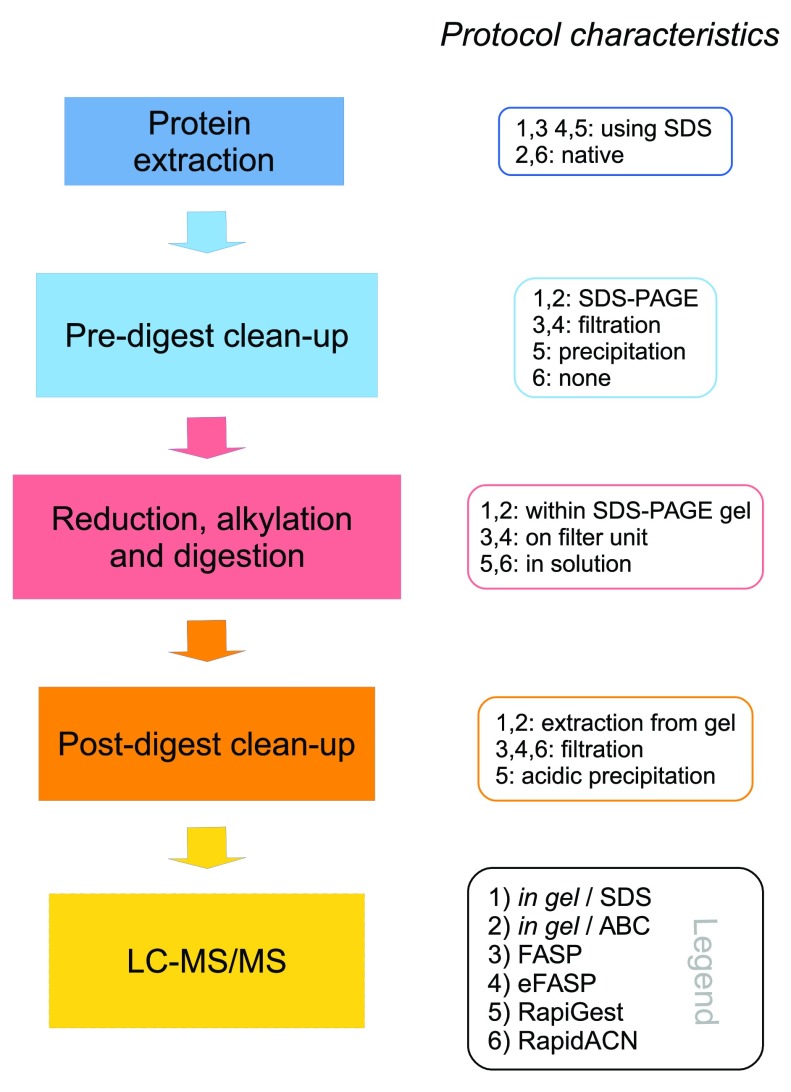
Characteristics of label-free sample preparation methods. **Left panel:** Schematic overview of the different steps in an LC-MS/MS sample preparation method. **Right panel:** Main characteristics of the protocols compared in this study. Detailled protocols are given in the Supplementary material. Supplementary protocol 1: In gel/SDS; 2: In gel/ABC; 3: FASP; 4: eFASP; 5: RapiGest; 6: RapidACN.

For this comparative study we selected an *in gel* digest method adapted from ^19^, conducted in combination with an SDS-based and native protein extraction, two *filter-aided* ( *FASP* (Filter Aided Sample Preparation) adapted from ^32^ and a recent enhancement termed *eFASP* adapted from ^33^, and two *in solution* procedures (RapiGest, adapted from ^22^, and RapidACN adapted from ^16^). Their characteristics are summarized in Figure 1. All procedures are given in lab-protocol format as Supplementary protocol 1 to Supplementary protocol 6. Please note that after the first version of this manuscript was published, an alternative sample preparation method by Erde *et al.* was also named eFASP which describes however an alternative protocol.

### *In gel* digestions

*In gel* digestions are popular sample preparation methods as they are convenient, and offer a simple way of protein pre-fractionation through gel slicing and removal of small or high molecular contaminants that could interfere with trypsin digestion. These approaches are compatible with multiple sample extraction buffers, can easily be combined with gel staining that does not interfere with protein digestion ^34– 36^, and thus provide a visual quality control over the protein sample. However, casting and running the gels render these protocols time consuming; hence the protocols are of relatively low throughput. In this study, we benchmarked *in gel* digestion in combination with both SDS-containing ( Supplementary protocol 1) and SDS-free protein extraction ( Supplementary protocol 2) ( *In gel*/SDS and *In gel*/ABC, respectively ^19^, Table 1). SDS-PAGE was however not used as a tool for pre-fractionation. In order to compare *in gel* methods with *filter-aided* and *in solution* digestion, the full mass range was processed and measured at once.

### Filter-aided sample preparation

The second set of assessed protocols involves digestion on filter units. These protocols are popular due to their flexibility and due to the fact that they facilitate a simple handling and require only a modest hands-on time ( ^~^3 hrs). The first protocol tested, FASP ^32^ involves a dual protease digest (Lys-C and trypsin), while the second *filter-aided* procedure (here called eFASP) is a stepwise-optimized version of FASP by Shevchenko and colleagues ^21^ that involves protein precipitation.

### *In solution* digestions

The final two protocols tested in this study perform protein digestion *in solution*. The first protocol is based on the proprietary, acid degradable detergent RapiGest (Waters ^37^), included in a protocol derived from Von der Haar *et al.* ^22^. This protocol involves protein precipitation, which renders the RapiGest procedure more laborious as compared to the second *in solution* protocol, termed RapidACN. This rather simple method is based upon a tryptic digest in acetonitrile that is combined with a filter-based sample cleanup ^9^. The RapidACN method requires the least number of handling steps and lowest hands-on time ( ^~^2 hrs per sample), overall facilitating the highest throughput among the tested procedures.

### Protein identification and compartment specificity

The six protocols, provided as detailed protocols in the Supplementary materials, were used to process an identical, full proteome sample of *Saccharomyces cerevisiae*. This single cellular eukaryote possesses a proteome of medium complexity (6,000–7,000 protein coding genes ^38^) and has served as a reference organism in many landmark proteome studies ^29, 39– 41^. Here, the use of yeast facilitated sampling from a single culture, bypassing the possibility of biological variability occurring between samples analyzed. However, once proteins are extracted, the here tested protocols are fully applicable to processing samples obtained from other species as well. To process the yeast pellets, the protocols were executed as close as possible to their original recipes (with unavoidable minor deviations highlighted in the Protocol section), both in complete replicates (= protocol triplicates), and in injection replicates for comparing the acquisition methods (= injection triplicates). Samples were analysed on a hybrid quadrupole time of flight (TripleTOF5600, AB/Sciex) mass spectrometer for DDA and SWATH acquisition, or on a hybrid quadrupole orbitrap mass spectrometer (QExactive, Thermo Scientific) for DDA acquisition. DDA database searches were conducted using Mascot (for TripleTOF5600 and QExactive, Matrix Science, ^42^) or ProteinPilot ^43^ (for TripleTOF5600, AB/Sciex), whilst SWATH data was processed with Skyline ^31^ and Spectronaut ^44^ (Biognosys) software.

It is noteworthy that in this study the analytical setup was adapted for quantification and not to maximize the number of protein identifications. This involved the injection of low amounts of sample (equalling 1 µg digest per protocol) to prevent column overload and considerable overrun of the dynamic range. Moreover, to allow a direct comparison of the protocols, data was recorded in single injections and samples were not pre-fractionated. This strategy yielded highly reproducible quantification results, achieving up to < 5% coefficient of variance (CV) values in label-free replicate injections for some protocols, as shown in Figure 4.

### Digest efficiencies

**Figure 2.**
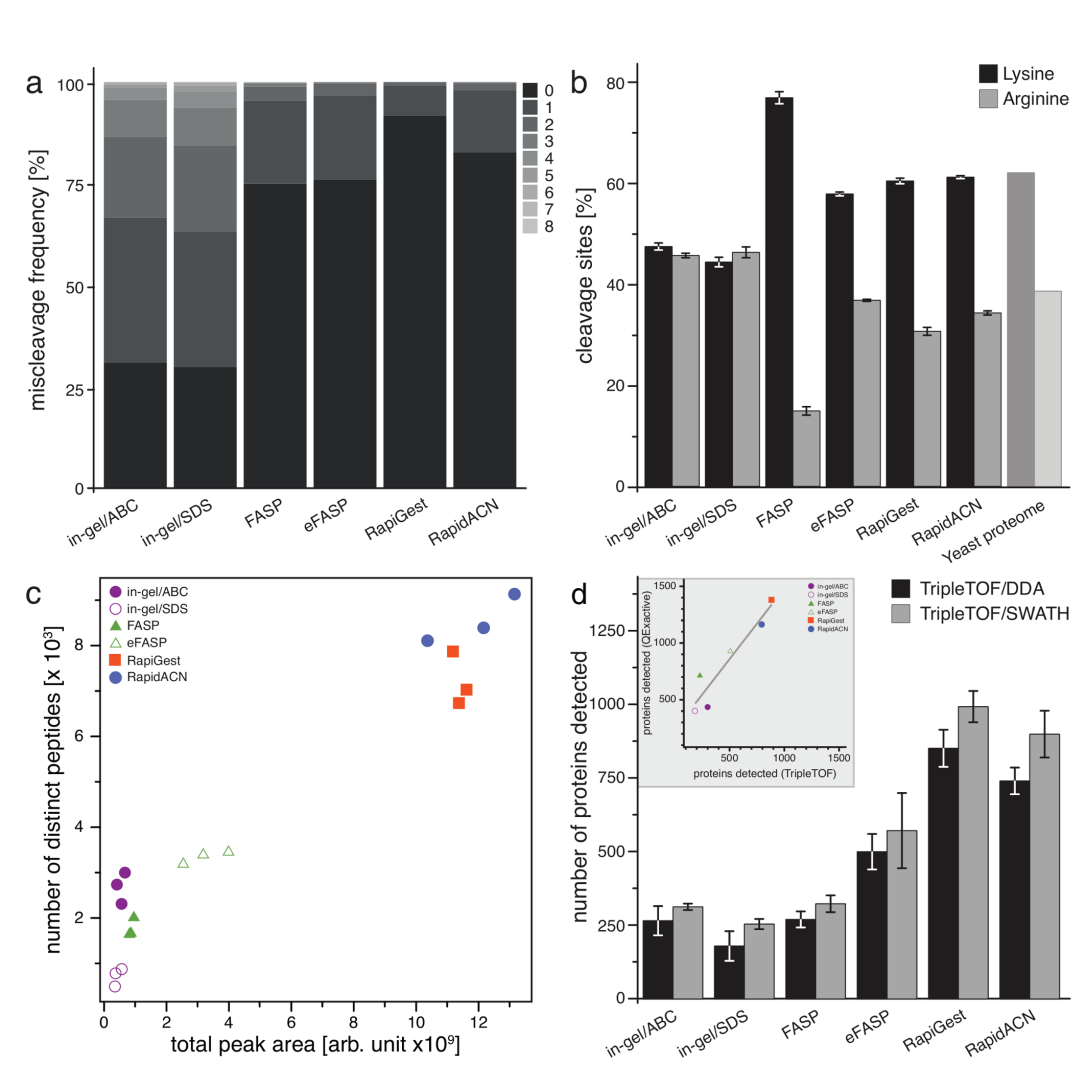
Protein identification in label-free sample preparations. ( **a**) **Proteolytic digestion efficiencies.** Trypsin or Lys-C/trypsin (FASP) digestion efficiencies expressed as relative occurrence of spectra that could be assigned miscleaved peptides (n = 3). ( **b**) **Amino acid specificity of proteolytic digestion.** Relative occurrence of identified peptides with C-terminal lysine or arginine, compared to the average frequency of these amino acids across all individual proteins identified (n = 3, Error bars = +/- S.D.) ( **c**) **Identified peptides differ per protocol, and correlate with the total peak area as recorded in a DDA experiment.** 18 samples derived from the same yeast culture were processed with six protocols in triplicates, and analyzed on a TripleTOF5600 instrument. The number of identified peptides correlates with the total peak area recorded, and indicates the highest identification rate in *in solution* digests, followed by *filter-aided*, and *in gel* procedures. ( **d**) **Detection of proteins by DDA or SWATH in a label-free experiment.** Samples were analyzed in triplicates both for DDA and SWATH acquisition on a TripleTOF5600 instrument, data was searched using paragon (DDA), and Spectronaut (SWATH). SWATH increased the number of detectable proteins in combination with the *in solution* protocols. *In solution* protocols RapidACN and RapiGest led to the detection of up to 1000 proteins in single injections, followed by FASP and eFASP, which gave rise to between 250 and 750 proteins, and *in gel* injections that yielded 300 proteins IDs. **Inset:** A comparison of protein IDs from the TripleTOF and QExactive platforms shows a linear correlation for the protocols investigated. Data was searched using Mascot (n = 3, Error bars = +/- S.D.).

As an indicator of the quality of tryptic digests, we first assessed the relative occurrence of partially cleaved peptides in data obtained from triplicate injections on the TripleTOF platform. All *filter-aided* and *in solution* protocols yielded reasonable digestion efficiencies as revealed by an analysis with both Paragon (AB/Sciex, Figure 2a) and Mascot (Matrixscience, data not shown) search engines. Both *in solution* and the eFASP procedure yielded arginine- and lysine cleavages in a similar ratio as found in the yeast proteome, with the lowest number of spectra assignable to missed cleavage tryptic sites found in the RapiGest dataset ( Figure 2a, and Figure 2b). In the fourth protocol (FASP), however, we found that the lysine cleavages overrepresented compared to arginine cleavages ( Figure 2b). This indicates that the presence of Lys-C in this protocol increased the overall digestion efficiency of lysine residues; however this may introduce a bias in (absolute) quantification experiments by overrating lysine over arginine peptides in quantification. With the employed *in gel* protocols we obtained a significantly higher number of spectra that corresponded to uncleaved peptides. As a further indicator of incomplete digestion, these protocols also gave a similar number of arginine and lysine peptides ( Figure 2a). Incomplete cleavage of peptides can render a sample preparation unsuitable for absolute quantification, but also for relative quantification, as the rate of cleavage may not be reproducible between replicates ^9^. For this reason, we consider the *in gel* protocols as employed (without prefractionation on the whole-proteome sample) to be potentially erroneous in protein quantification and identification, and excluded the data from the assessment of protein quantification quality. This result however does not exclude the possibility that on other samples, in combination with gel slicing (geLC-MS), or with modified *in gel* protocols, acceptable cleavage efficiencies are achieved, and thus, this result should not be interpreted as a critique of *in gel* methods in general.

### Protein identification

The number of detected peptides correlated with the sum of recorded total peak area, confirming that the instrument was operating within its dynamic range ( Figure 2c). The yield of detected peptides ( Figure 2c) and proteins ( Figure 2d) revealed different performance of the tested protocols. For both data dependent (DDA) and SWATH acquisition, the two *in solution* protocols (RapiGest and RapidACN) gave the highest number of detectable peptides and proteins. Filter-based FASP and eFASP protocols ranked in the middle range, whilst a significantly lower number of proteins were detected from the *in gel* digests. Of note, SDS-based compared to native protein extraction increased the number of membrane protein detections in the *in gel* procedure, but in total a higher number of peptides were obtained in the natively extracted samples. To exclude the possibility that these results were platform specific, we injected the same samples on a QExactive mass spectrometer, operating with a different HPLC system and column (Dionex Ultimate 3000; 2 µm particle size C18, 75 µm i.d. × 50 cm column, see methods section). The number of protein IDs obtained with the two platforms correlated linearly, indicating that the ID performance of the tested protocols is platform independent ( Figure 2d, Inset). Additionally, we tested to what extent injecting higher amounts of sample or pre-fractionation would increase the number of identifiable proteins. Single injection of 10 times the RapidACN sample increased the number of identifiable proteins by 34% to 1550 (QExactive), while high-pH RP HPLC pre-fractionation of a RapidACN digest led to the identification of 2800 proteins (TripleTOF). Similar tendencies were observed with the other protocols as well, indicating that when combined with sample pre-fractionation, all protocols and both platforms are suitable for ID-optimized experiments, as addressed in other studies.

To be able to compare data dependent (DDA) and data independent (DIA) acquisition in terms of protein detection, we then analysed the samples using SWATH mode. Overall, when setting the highest quality threshold on SWATH-detected peptides (Spectronaut Q value < 0.01), SWATH and DDA detected a comparable number of proteins for the *in gel* and FASP procedures. However, SWATH outperformed DDA in the samples with higher peptide content, RapiGest and RapidACN, leading to a modest but consistent increase in protein detection numbers ( Figure 2d).

### Performance of sample preparation methods in covering the variety of the proteome

**Figure 3.**
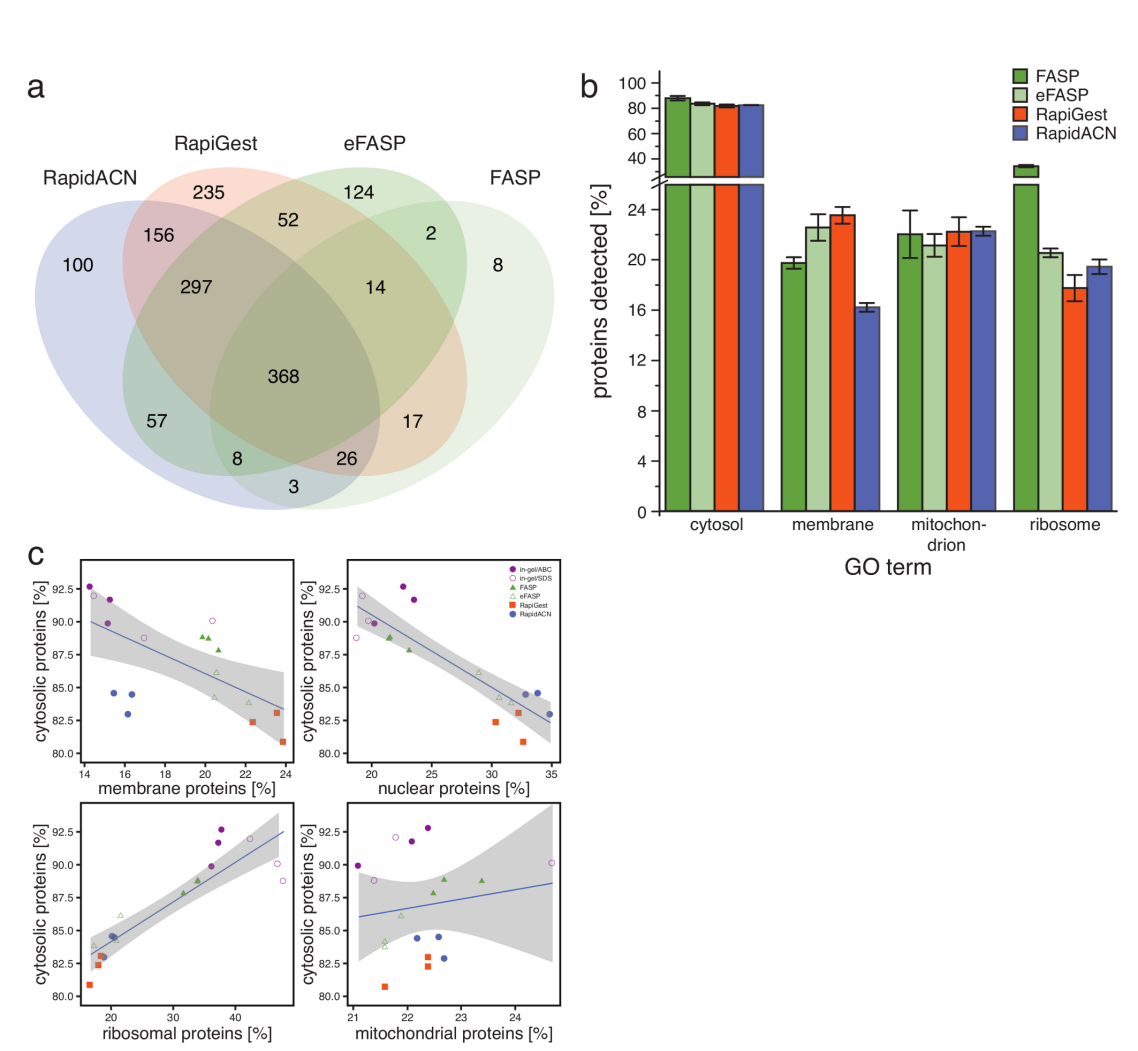
Protocols cover cellular compartments differently. ( **a**) **RapiGest and eFASP cover a unique space in the proteome.** Identified proteins were visualised in a Venn diagram, excluding the *in gel* protocols. The RapiGest procedure yielded most unique IDs, followed by eFASP and RapidACN (n = 3). ( **b**) **SDS-containing protocols are best suited for the extraction of membrane proteins.** For the analysis of annotated functions in each protocol, selected GO terms were expressed as percentages of identified proteins. While cytosolic proteins were not enriched in any protocol, membrane proteins were preferentially detected in the SDS-containing protocols. (n = 3, Error bars = +/- S.D.) ( **c**) ***Filter-aided* sample preparations yield a balanced representation of the proteome.** The identified proteins were plotted against the percentage of proteins annotated by the GO term cytosol, in order to illustrate the similarity of extraction properties. The protocol properties required for efficient extraction of membrane and nuclear proteins is inversely correlated with the extraction efficiency for cytosolic proteins, while there is a positive correlation with ribosomal proteins.

Next we used the TripleTOF/DDA data to assess whether the protocols covered a similar set of proteins. A subset of 368 proteins overlapped between protocols 3, 4, 5 & 6 (all *filter-aided* and *in solution* protocols), while the *filter-aided* protocols ( Supplementary protocol 3 and Supplementary protocol 4) overlapped for 479 proteins, and the *in solution* protocols ( Supplementary protocol 5 and Supplementary protocol 6) for 915 proteins ( Figure 3a). Due to high occurrence of uncleaved peptides, which may affect protein identification, the *in gel* methods are omitted from this illustration. Indeed, the proteins identified in the *in gel* samples were to > 95% covered by *filter aided* and *in solution* methods as well (data not shown). All other protocols covered specific sets of proteins. RapiGest yielded the highest absolute number of unique IDs, while eFASP provided the highest percentage. Hence, in targeted proteome studies, sample preparation with different protocols might be considered in order to increase the probability of quantifying the desired target.

We next assessed whether these differences correlated to the coverage of cellular localisations. The tested protocols gave high coverage of the GO term cytosol, and performed equally on the mitochondrial proteome ( Figure 3b, see Supplementary Figure S2 for a complete overview of GO terms). However, different results were obtained for membrane proteins. The lowest relative content of membrane proteins was obtained for those protocols that extract proteins under non-denaturing conditions, namely RapidACN and *in gel/ABC*. Conversely, most membrane proteins were detected in the detergent-rich protocols, eFASP and RapiGest. Overall, FASP and eFASP yielded the most balanced representation of both the membrane and cytosolic fraction, while RapidACN data exhibited the strongest bias towards cytosolic and against membrane proteins ( Figure 2c).

**Figure S1.**
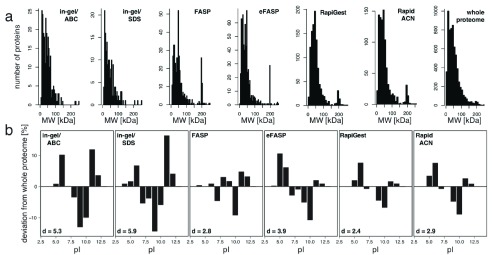
Bias of protein size and pI in sample preparation. ( **a**) **The spectrum of protein sizes is well covered for all protocols examined.** The number of identified proteins was plotted against the theoretical molecular weight (MW) for each protocol investigated. Although some protocols yielded higher identifications than others, the MW range was highly reproducible for all of them when comparing against the whole yeast proteome. ( **b**) **Different representations of protein charges.** When comparing to the distribution of theoretical protein pI values of the whole proteome, all investigated protocols showed an under-representation of proteins with a pI of 10. When expressing the total deviation as deviation score d, RapiGest, FASP and RapidACN score best. The d values were calculated as the sum of all differences in % compared to the theoretical proteome occurrence multiplied by 0.1.

**Figure S2.**
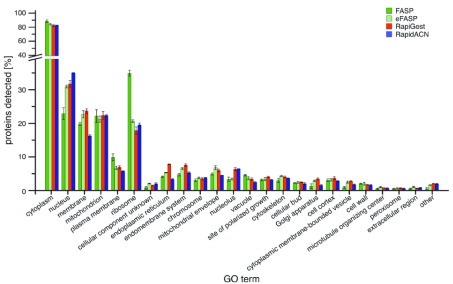
Distribution of GO terms associated with identified proteins. The percentage of GO annotations for each protocol is shown, allowing multiple annotations for individual proteins (n = 3, Error bars = +/- S.D.).

Finally, we tested whether the protocols covered the proteomic mass range and charge state equally. The proteomic mass range was similarly represented by all protocols with a slight positive bias towards large proteins in all protocols ( Supplementary Figure 1a). The procedures, however, differed in the representation of proteins with a certain isoelectric point (pI). The best representation of the proteome pI distribution was obtained with RapiGest (deviation coefficient (d) = 2.4), followed by FASP (d = 2.8) and RapidACN (d = 2.9) ( Supplementary Figure 1b). *In gel* procedures scored least as they were negatively biased towards neutral proteins, and achieved a lower d value of 5.3 or 5.9 for *in gel/ABC* or *in gel/SDS*, respectively.

### Label-free quantification

**Figure 4.**
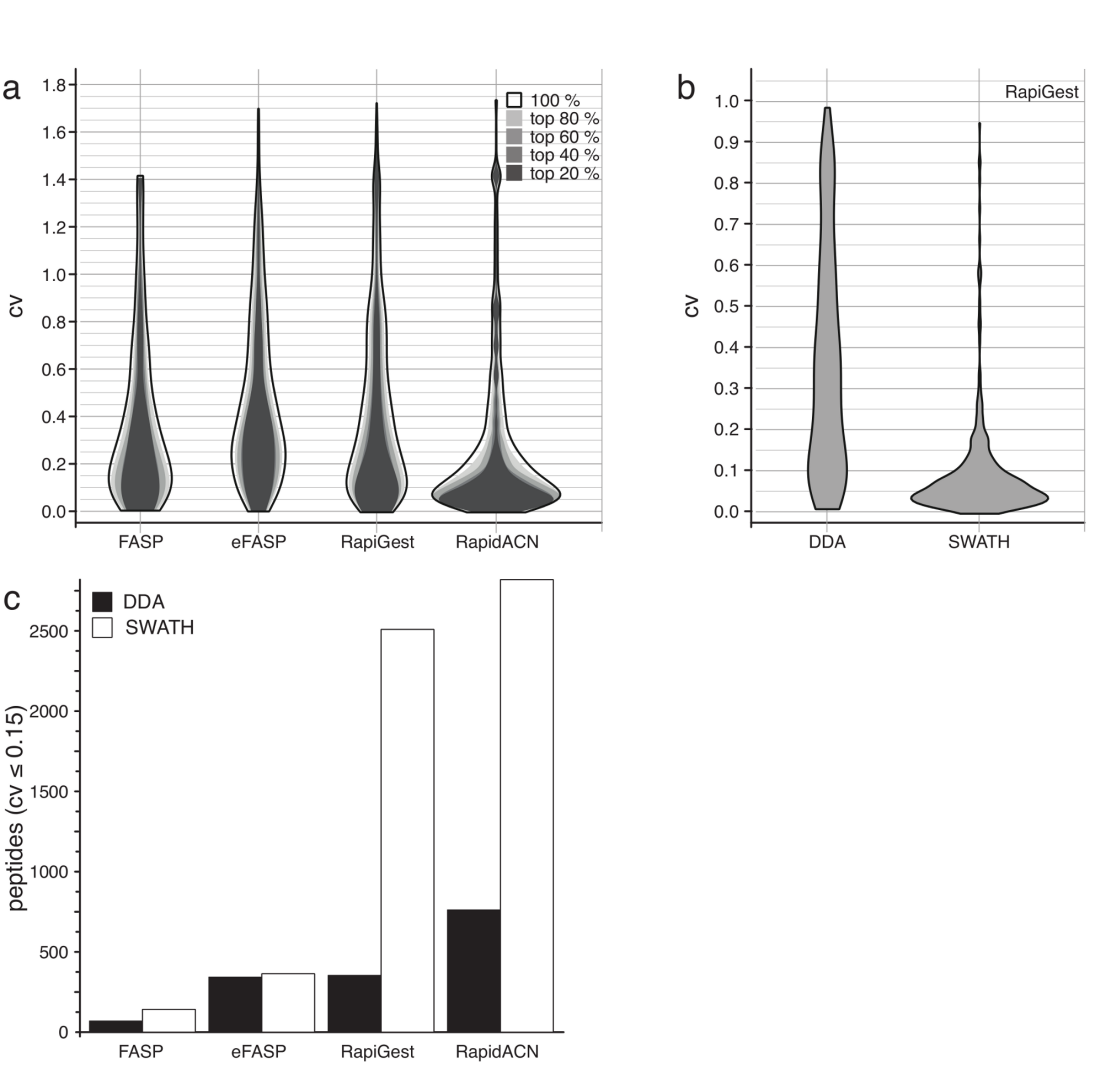
*In solution* digestion leads to stable results in label-free proteomics. ( **a**) **The distribution maximum of coefficients of variation (CV) of the selected protocols varies between 0.075 and 0.2.** CV values obtained for protocol triplicates are shown as two-dimensional distribution histograms (‘violin plots’). Quantification in DDA experiments was consistent over the dynamic range, as CV values only marginally changed when filtering by peptides according to their abundance (80%, 60%, 40% or 20%). CV likelihood maxima of all protocols were below 20%, while RapidACN led to the most reproducible results (CV = 7%) (n = 3). ( **b**) **Stability in a quantification experiment is improved by data-independent acquisition.** CV values for the same set of peptides measured with SWATH and DDA using the RapiGest protocol, as shown in a two-dimensional distribution histogram. Whereas there was a high signal variation in DDA acquisition, the variation could be largely reduced in SWATH acquisition. ( **c**) ***In solution* protocols yield the highest number of peptides suitable for label-free quantification.** The number of peptides with a CV < 0.15 as determined in DDA and SWATH acquisitions. The number of highly reproducible peptides was lower for FASP and eFASP, and SWATH acquisition did not improve the performance in combination with these methods. *In solution* protocols on the other hand did yield a maximum of about 2500 high-quality peptides using SWATH.

Next, we compared the protocols for their consistency in label-free quantification. As illustrated in Figure 2c, the number of identified peptides correlated with the sum of total peak area recorded, hence all procedures in principle lead to quantitative results. To be able to compare the protocols, we expressed the variation of signal intensities obtained from replicate sample preparations as coefficient of variation (CVs), and we plotted the frequency of CVs in two-dimensional distribution histograms (‘violin plots’, Figure 4a). DDA acquisition resulted in a CV maximal likelihood of 20% for eFASP, FASP and RapiGest. Although most peptides showed a variation of this magnitude, it is worth noting that there was a considerable spread of CVs in all three protocols, with some peptides showing as much as 140% variation. By far the highest signal reproducibility with a CV maximal likelihood of 7% was obtained with the RapidACN protocol ( Figure 4a), indicating best suitability of this protocol in label-free quantification.

**Table 1.** Sample preparation methods and their performance. Summary of the main characteristics of the sample preparation methods investigated.

Protocol Name	*In gel/SDS*	*In gel/ABC*	FASP	eFASP	RapiGest	RapidACN
**Reference**	Based on Kaiser *et al.*, 2008	Based on Kaiser *et al.*, 2008	Wisniewski *et al.*, 2009	Shevchenko *et al.*, 2012	Waters (UK), based on von der Haar *et al.*, 2007	Bluemlein *et al.*, 2011
**Digest**	*In gel*	*In gel*	*Filter-aided*	*Filter-aided*	*In solution*	*In solution*
**Lysis**	4% SDS, DTT	0.05 M ABC	4% SDS, DTT	1% SDS, EDTA	2% SDS, β-ME, EDTA	0.05 M ABC
**Protein** **precipitation**	-	-	-	Yes	Yes	-
**Additive for** **digestion**	-	-	Urea	nOGP	RapiGest SF	Acetonitrile
**Use of ﬁlter** **units**	-	-	for digest	for digest	-	for sample clean-up
**Protease**	Trypsin	Trypsin	Lys-C + Trypsin	Trypsin	Trypsin	Trypsin
**Total time**	29 hrs	29 hrs	32 hrs	30 hrs	30.5 hrs	26 hrs
**(of which** **digest)**	(16 hrs)	(16 hrs)	(20 hrs)	(16 hrs)	(18 hrs)	(18 hrs)
**Hands on time**	5 hrs	5 hrs	3 hrs	3 hrs	3.5 hrs	2 hrs
**Consumable** **costs**	<0.5	<0.5	10.75	1.8	1 (=2 £)	1.95
**Overall** **throughput**			★	★	★★	★★★
**Total protein** **IDs**				★	★★★	★★
**membrane** **proteins**			★★★	★	★★	
**CV in label-free** **quantification**			★		★★	★★★
**Comments**	+ Cost effective + Matrix independent	+ Cost effective + Matrix independent	+ Best pI coverage + Cell compartments	+ Lipid removal	+ High ID numbers + Low CVs + Good coverage of membrane proteins	+ Highest throughput + Lowest CVs + Highest No of quantiﬁable peptides
	- Low ID numbers	- Low ID numbers	- Dual protease digest	- Traces of nOGP in sample - High CV	- Dependent on proprietary reagent - Traces of detergent may reside	- Low number of membrane proteins

Next, we counted the number of precisely quantified peptides, defined as peptides with a CV < 15%. Also in this measure, the RapidACN procedure outperformed the other methods, while RapiGest, and eFASP performed second and third best, respectively ( Figure 4c). Not covered in this benchmark is the performance of the individual protocols in repeated sample preparation over longer periods, i.e. weeks to months. This might be required for particular sets of samples that can not be stored without a protease digest, yet require sampling on different days to address a specific biological question.

Finally, we tested whether SWATH analysis improved label-free quantification. Comparing the CV distribution of peptides detected both in DDA and SWATH data using the RapiGest protocol ( Figure 4b), we discovered a much more focussed CV distribution around a maximal likelihood of 5% in SWATH, compared to a maximal likelihood of 20% in DDA mode. When counting the number of precisely quantified peptides (CV < 0.15), on the TripleTOF instrument SWATH led to an increase of up to a factor of two and five for RapidACN and RapiGest, respectively ( Figure 4c). Hence, SWATH acquisition greatly improved the CV stability with label-free acquisition, the result of which is that a substantial number of peptides were precisely quantified.

LC-MS/MS spectral information Protein pilot (.group) files containing spectral data acquired with the 6 protocols tested in this study. Data files contain both full technical replicates (= protocol replicates) for evaluation of the protocol performance, and LC-Ms/MS replicates (= injection replicates) for evaluating the performance of the LC-MS/MS platform and acquisition strategy"

## Discussion

Stable isotope labelling is a popular and reliable strategy in quantitative proteomics, yet has limitations that arise from an increased analyte load in the precursor ion (MS1) space, and the way standards are produced or incorporated. For instance, targeted protein quantification using AQUA peptides ^45^ achieves absolute quantification through comparison between the peak areas of light and chemically synthesized heavy-isotope labelled peptides of known concentration. However the costs for such peptides limits the number of proteins quantifiable ^7, 45^. An alternative strategy is the non-targeted chemical labelling of proteins and peptides with isobaric tags (i.e. iTRAQ, TMT), facilitating multiplexing of proteome samples and providing relative simultaneous quantification of labelled peptides ^8, 46^. However, frequent co-selection of the reporter ions reduces both the accuracy and precision of quantification ^47, 48^. Such a problem is circumvented when metabolic incorporation of isotope-labelled amino acid residues (i.e. SILAC ^49^, or recent extensions like for instance NeuCODE which is based on different nuclear masses dependent on the isotope combination integrated ^50^), is used to create isotope-labelled standards *in vivo*. However, this approach is limited to heterotrophic species that consume lysine and arginine from the culture medium, and is in practice limited to tissue culture as the attempt to introduce labelling in animal models becomes extremely expensive ^51^.

Label-free experiments circumvent the use of isotope labelled standards, thus are not affected by the above-mentioned limitations. As such, label-free experiments are ideal complements when isotope labelling becomes a limitation. However, the label-free method or strategy lack possibilities to correct for selective sample loss, and hence are more sensitive to variations in sample preparation and instrument performance. The protocols employed consequently require more rigorous validation.

### *In gel* digests

Our comparison starts with a classic *in gel* digestion method ^19^, which is tested in combination with SDS-containing- and SDS-free protein extractions ( Supplementary protocol 1 and Supplementary protocol 2). These popular cost-effective procedures are based on the principle that a protein sample is denatured and separated on an SDS-PAGE gel prior to reduction, alkylation and protease digestion that are conducted within the gel matrix. The gel fulfils the function of sample clean up, as it removes positively charged contaminants as well as large macromolecules (i.e. nucleic acids) and small chemical compounds, and is very robustly applied to a large variety of sample types. Furthermore, the excision of individual bands or mass ranges make *in gel* digestions attractive wherever a simple sample pre-fractionation is required. Proteome pre-fractionation *in gel* (geLC-MS) has resulted in a significant proteome depth and dynamic range in studies were > 5000 distinct proteins were confidently identified and quantified ^52, 53^. Moreover, *in gel* digests have proven ideal when gel bands resulting from individual proteins are to be identified (i.e. for studying protein complexes). In the present study however, we did not make use of sample pre-fractionation. In order to achieve comparability with the other protocols, the full mass range was processed for the digest (see Methods section, and Supplementary protocol 1 and Supplementary protocol 2). This treatment led to a full representation of the proteomic mass distribution ( Supplementary Figure S1). Under these circumstances however, the classic *in gel* protocol applied proved the least suitable method for label-free quantification. The protocol was the most time consuming, yet yielded a significant number of miscleaved peptides, and we detected the lowest number of proteins and peptides in total. Differences between SDS-free and SDS-containing sample extraction affected the relative content of membrane proteins identified, which was higher in the latter, whereas the native (SDS-free) extraction resulted in a higher number of proteins identified in total. This result should however not be interpreted as a general critique on *in gel* methods for other applications, as in combination with protein pre-fractionation (gel-slicing), they have proven well as suitable sample preparation methods in ID experiments ^52, 53^.

### Filter-aided sample preparation

The dependence on filter units in the two tested *filter-aided* sample preparation procedures, FASP ^32^, and one of its recent extensions (here called eFASP ^21^), increases the material costs, but has advantages for sample handling and throughput. Indeed, handling of the first protocol, FASP, was efficient and achieved a reasonable throughput with modest hands-on time ( Supplementary protocol 1). In protein identification, FASP achieved the highest relative amount of detected membrane proteins. Hence, this protocol might be an ideal choice when membrane proteins are to be studied.

FASP was the only protocol in this study where digestion was carried out using a combination of proteases, Lys-C and trypsin. Similar to previous reports ^54^, we observed that the addition of Lys-C increased the relative digestion efficiency. However, this resulted in an over-representation of lysine over arginine containing peptides, which may lead to bias in cases where this protocol is used in an absolute quantification experiment. In label-free quantification, FASP performance compared to the other protocols, was average both in the number of precisely quantified peptides and in the CV values obtained for replicative sample preparations. It is important to mention in this context that the performance of FASP procedures is dependent on the filter units that are available from different manufacturers, however the exact filter unit used in the original FASP paper ^32^ is no longer available. In this study we have chosen Amicon Ultra-0.5 3k for both FASP based protocols as used in eFASP by Shevchenko *et al.* ^21^, as their cut-off rate (3kDa) is the closest to the addressable mass range of the SWATH acquisition (400–1200 m/z). Further work from Wisniewski *et al.* demonstrated that also larger cut-off rates up to 50k are suitable in combination with the FASP protocol, and can improve the identification rate of larger proteins and peptides ^55^. Moreover, in difference to the other protocols tested in this study, the tryptic digest in FASP is conducted in a very high concentration of urea. A simple protocol adaptation to influence the tryptic digest could thus be to change the buffer conditions, e.g. to a buffer as used in eFASP ^21^ ( Supplementary protocol 4).

The second *filter-aided* protocol, eFASP, represents a stepwise optimisation of FASP, and contains several alterations compared to its predecessor ^21^ ( Supplementary protocol 4). The protease digest is performed using trypsin only, and the protocol includes a lipid removal step and uses *n*- *octyl*- d-glucopyranoside (nOGP) as the detergent in sample preparation. The latter might be regarded as an undesirable addition to the sample, as nOGP can interfere with electrospray ionisation. Indeed, despite all washing steps, we could detect traces of nOGP in the MS/MS spectra, and the collection of MS data was reduced at the time a nOGP sodium adduct eluted (data not shown). Despite this, the modifications made for eFASP clearly improved the performance in protein and peptide identification. However, in our hands, they did not improve the precision in label-free quantification, therefore the performance of FASP and eFASP in this measure was comparable ( Figure 4). Hence, the main advantage of eFASP over FASP lies in improvements in protein identification and proteome coverage. Please note that the protocol by Shevchenko *et al.* differs from a recently published protocol by Erde *et al.*, also termed eFASP

### *In solution* digestion

The first method tested ( Supplementary protocol 5) is based upon the commercial reagent RapiGest SF (3-[(2-methyl-2-undecyl-1,3-dioxolan-4-yl)methoxy]-1-propanesulfonate ^37^ (Waters)), an anionic detergent which is depleted from the sample through acidic cleavage. The established protocol ^22^ contains a step for lipid removal and a precipitation step that renders this procedure more laborious compared to the FASP and RapidACN protocols. However, as it does not involve any filter unit, it was most economic in terms of material costs per sample if one disregards the *in gel* protocols. Moreover, it yielded the highest number of protein and peptide IDs, and it detected the highest absolute number of membrane proteins. In label-free quantification, it scored third best in the average CV for DDA, and second best in combination with SWATH acquisition. Expressed in absolute quantities, this method yielded the second-highest number of precisely quantified peptides. Thus, the RapiGest protocol is a versatile and economic method that may represent the optimal choice in many applications. The only inexplicable issue with this protocol was related to the inefficiency of RapiGest degradation and precipitation in a small subset of samples. Thus care must be taken to avoid detergent; injection in the LC-MS/MS setup.

The second *in solution* protocol (termed RapidACN ^9^, Supplementary protocol 6) is detergent-free and based on acetonitrile in sample processing and proteolytic cleavage, followed by clearing samples from high-molecular weight contaminants by a final filtration step. As this protocol is based on a native protein extraction, it identified - in relative terms - the lowest number of membrane proteins. Moreover, as it does not contain an intensive pre-digest sample treatment, functionality of this protocol may omit tissue where such a forefront clean up is mandatory. Despite these limitations, RapidACN performed best in the metric most crucial for robust label-free quantification, a low CV value in replicate sample digests and injections. Moreover, compared to the other tested methods, RapidACN was simplest in handling, required the least processing steps and only minimal hands-on time (~2 hrs), while yielding the second highest number of protein and peptide detection both in DDA and SWATH acquisition methods. Hence, RapidACN might be the most suitable solution for a label-free experiment when the focus is not to quantify membrane proteins, or to analyze tissue that requires extensive clean up.

### Data-dependent versus data-independent acquisition

We chose to perform major parts of this study on a TripleTOF5600 instrument (AB/Sciex), in order to compare data-dependent acquisition (DDA) with data-independent acquisition (DIA). DIA is believed to be advantageous for label-free quantification, as it is less affected by run to run variations, and as MS2 data is reconstructed in chromatograms that resemble selective reaction monitoring (SRM) ^17^. Therefore, this technique appears a desirable choice for the label-free analysis of biological time series, that require many samples (replicates over many time-points) to be compared ^15^. The design of the TripleTOF5600 quadrupole allows precursor ion selection in a rectangular rather than a Gaussian mass selection window as in other instruments, reducing the co-selection of peptides falling in the adjacent mass windows ^23^. In a workflow termed SWATH, the mass range from 400 to 1200 m/z (in this study: 400–1250 m/z) is scanned in 25 Da windows, and the merged data used to reconstruct spectral (MS ^2^) m/z chromatograms ^17^. Processing SWATH data with Spectronaut (V. 3.0.337, Biognosys), we compared the performance of DDA with SWATH in protein detection and label-free quantification. In samples with low peptide content, the number of detected proteins with DDA and SWATH was comparable. However, in the *in solution* protocols that led to highest IDs, SWATH acquisition gave a slight but significant advantage in terms of peptides detected. This indicates that this approach is advantageous in protein detection when coupled with complex matrices. In contrast, SWATH was however clearly advantageous in label free quantification. The strongest improvement for SWATH over DDA acquisition was observed when it was used in conjunction with the RapiGest protocol ( Supplementary protocol 5), where the number of precisely quantified peptides increased by a factor of five, followed by the combination with RapidACN ( Supplementary protocol 6), where this measure doubled ( Figure 4c). Of note, SWATH employed in combination with the RapidACN, resulted in an average CV below 5%, representing a superior value obtained in a label-free experiment. These improvements mainly resulted from a more precise quantification of peptides in the mid to high abundance range, whereas there was no increased improvement in quantification of low abundant spectra. We assume that this difference could be further optimized by improving the SWATH peak selection algorithms, as noise in the low abundance window results from occasional misassignment of fragment ions to precursors.

## Conclusions

By facilitating label-free quantification, second-generation proteomics techniques enable flexible proteomic workflows. As the protocols cover different sets of proteins and cellular compartments, the main determinant to select the best suitable method and workflow remains the biological question and the set of proteins to be addressed. Despite this, sample preparation methods differ in precision, sensitivity and throughput. Under the conditions of this benchmark, and under the conditions in our laboratory, a combination of *in solution* digestion protocols RapiGest or RapidACN with SWATH acquisition yielded optimal results for a label-free proteomics experiment. Achieving reliable quantification at reasonable numbers of detected proteins, label-free quantitative proteomics represents a suitable alternative to isotope labelling in addressing a series of biological problems.

## Figures and Tables

**Table S1.** Rolling Collision Energy settings of the TripleTOF platform.

Charge	Slope	Intercept
Unknown	0.044	4
1	0.0575	9
2	0.044	4
3	0.05	3
4	0.05	2
5	0.05	2

(CE=(slope)*(m/z)+intercept

## Supplementary materials

**RAW data:** The .wiff files of the DDA and DIA acquision used in Figure 2– Figure 4 are downloadable as Supplementary Data. Supplementary table ST1 lists the different file names.

## Supplementary protocol 1: *In-gel* digestion in combination with SDS extraction

(Adapted from Kaiser, P, Meierhofer, D, Wang, X, Huang, L. Tandem affinity purification combined with mass spectrometry to identify components of protein complexes. Methods Mol. Biol. 2008, **439**: 309–326)

### Materials

***Solutions and reagents***

Lysis buffer: SDT buffer (4% SDS, 100 mM Tris/HCl pH 7.6, 0.1 M Dithiothreitol (DTT, Melford Cat No. MB1015)

ABC: 0.05 M Ammonium bicarbonate (Fluka Cat. No. 40867-50G-F) in water

DTT: 0.09 M Dithiothreitol (Melford Cat No. MB1015)

IAA: 0.1 M Iodoacetamide (Sigma Cat. No. I1149-5G) in ABC

Sequencing Grade Modified Trypsin: Stock 0.2 µg/µL (Promega Cat. No. V5111)

UPLC/MS grade water

FA: Formic acid

ACN: Acetonitrile

30% Acrylamide/0.8% Bis-acrylamide (Protogel, Geneflow Limited Cat. No. EC-890)

10% Ammonium persulfate (APS, Sigma Cat. No. A3678-25G)

10% Sodium dodecyl sulfate (SDS, Melford Cat. No. S1030)

1.5 M Tris/HCl pH 8.8

0.5 M Tris/HCl pH 6.8

BCA Protein assay kit (Pierce Cat. No. 23225)

***Equipment***

Protein Gel Casting Stand (BioRad)

Acid-washed glass beads (Sigma G8772-500G)

Protein LoBind tubes (Eppendorf Cat. No. 0030 108.094)

Fast Prep-24 instrument (MP Biomedicals)

Refrigerated bench top centrifuge

Spectrophotometer

Ultrasonic tank (Langford Electronics Limited)

Wet chamber

Vacuum concentrator centrifuge

### Method

***1 Sample lysis***

- Add 200±10 mg glass beads and 200 µL lysis buffer to 30 mg cells (wet weight, equaling 10 OD _600_ units of yeast cells)
- Vibrate the cell suspension in the Fast Prep-24 instrument (4°C, settings: 6.5 Ms ^-1^, 20 sec). Repeat this step 2 times with a 5 min interval, keep samples on ice between intervals
- Centrifuge at 16,000 × *g* for 1 min to sediment the cell debris
- Incubate for 3–5 min at 95°C
- Centrifuge at 16,000 × *g* for 1 min
- Transfer the supernatant to a new reaction tube
- Incubate for 5 min in an ultrasonic tank to reduce viscosity

***2 Protein quantification***

- Use BCA assay to determine the protein concentration according to the manufacturer’s instructions

***3 Sample processing***

**3.1 Protein separation by SDS-PAGE**

- Cast separating (15% acrylamide/bisacrylamide) and stacking gels (4%) according to Laemmli (Laemmli, UK. Cleavage of structural proteins during the assembly of the head of bacteriophage T4. Nature. 1970, **227**, 680–685)
- Add SDS-PAGE loading buffer to a lysate aliquot containing 50 µg protein and incubate at 95°C for 5 min to denature proteins
- Perform electrophoresis for 20 min at 100 V to allow the proteins to enter the separating gel
- Excise the sample from the gel in a single slice

**3.2 Excision and *in-gel* digestion of protein bands**

- Cut the gel slice into small pieces (1 mm) and place into a new reaction tube
- Add 100 µL (or enough to cover) 25 mM ABC/50% (v/v) ACN and vortex for 10 min
- Centrifuge at 16,000 × *g* for 30 sec and remove and keep the supernatant using a gel-loading micropipette tip. Repeat this step for 2 or 3 times
- Evaporate the solvents in a vacuum concentrator centrifuge (approximately 20 min)
- Add 50 µL (or enough to cover) 10 mM DTT in 25 mM ABC to the dried gel pieces
- Vortex and centrifuge at 16,000 × *g* for 30 sec
- Incubate with the reductive solution at 56°C for 1 h
- Remove the supernatant and add 50 µL (or enough to cover) 50 mM IAA to the gel pieces. Vortex and centrifuge at 16,000 × *g* for 30 sec
- Incubate with the alkylation solution in the dark for 30 min at room temperature, with occasional vortexing. Centrifuge at 16,000 × *g* for 30 sec
- Remove the supernatant. Add ~100 µL 25 mM ABC to the gel pieces. Vortex for 5 min and centrifuge at 16,000 × *g* for 30 sec
- Remove the supernatant and add ~100 µL (or enough to cover) 25 mM ABC/50% (v/v) ACN to dehydrate the gel pieces. Vortex for 5 min and centrifuge at 16,000 × *g* for 30 sec. Repeat this step
- Evaporate the solvents from the gel pieces in a vacuum concentrator centrifuge (approximately 20 min)
- Add 10 µL of trypsin (10 ng/µL) to the dried gel pieces and incubate for a few min to allow rehydration
- Add 25 µL 25 mM ABC (or sufficient volume to cover the gel pieces), vortex for 5 min, centrifuge at 16,000 × *g* for 30 sec and incubate at 37°C overnight in a wet chamber
- Centrifuge at 16,000 × *g* for 30 sec. Add 10 µL water, vortex for 10 min and centrifuge at 16,000 × *g* for 30 sec
- Transfer the tryptic peptides (aqueous extraction) into a new reaction tube
- Add 30 µL (or enough to cover) of 50% (v/v) ACN/5% (v/v) FA to the gel pieces, vortex for 10 min and centrifuge at 16,000 × *g* for 30 sec. Combine the supernatants of this and the previous step. Repeat this step once more
- Add 10 µL 100% (v/v) ACN to the gel pieces, vortex for 5 min and centrifuge at 16,000 × *g* for 30 sec. Combine with previous extractions
- Centrifuge the tryptic peptide mix at 16,000 × *g* for 30 sec and evaporate solvents in a vacuum concentrator centrifuge (approximately 2 hrs)
- Re-suspend the peptides in 50 µL 5% ACN/0.1% FA to obtain a final concentration of 1 µg/µL
- Aliquot and store tryptic peptides at -80°C

**Compared to the original protocol the following changes were made:**

- 4% SDS and 0.1 M DTT were added to the lysis buffer
- The lysis of yeast cells was performed using Fast Prep at 6.5 Ms ^-1^, 20 sec. This step was repeated 3 times with a 5 min interval on ice in between runs
- An incubation at 95°C for 5 min was performed to achieve a complete lysis of cells
- Sonication was performed to reduce viscosity of the sample

## Protocol 2: *In-gel* digestion in combination with protein extraction in ammonium bicarbonate (ABC)

(Adapted from Kaiser, P, Meierhofer, D, Wang, X, Huang, L. Tandem affinity purification combined with mass spectrometry to identify components of protein complexes. Methods Mol. Biol. 2008, **439**: 309–326)

### Materials

***Solutions and reagents***

Lysis buffer: 0.05 M Ammonium bicarbonate (Fluka Cat. No. 40867-50G-F) in water

ABC: 0.05 M Ammonium bicarbonate (Fluka Cat. No. 40867-50G-F) in water

DTT: 0.09 M Dithiothreitol (Melford Cat No. MB1015)

IAA: 0.1 M Iodoacetamide (Sigma Cat. No. I1149-5G) in ABC

Sequencing Grade Modified Trypsin: Stock 0.2 µg/µL (Promega Cat. No. V5111)

UPLC/MS water

FA: Formic acid

ACN: Acetonitrile

30% Acrylamide/0.8% Bis-acrylamide (Protogel, Geneflow Limited Cat. No. EC-890)

10% ammonium persulfate (APS, Sigma Cat. No. A3678-25G)

10% Sodium dodecyl sulfate (SDS, Melford Cat. No. S1030)

1.5 M Tris/HCl pH 8.8

0.5 M Tris/HCl pH 6.8

BCA Protein assay kit (Pierce Cat. No. 23225)

***Equipment***

Protein Gel Casting Stand (BioRad)

Acid-washed glass beads (Sigma G8772-500G)

Protein LoBind tubes (Eppendorf Cat. No. 0030 108.094)

Fast Prep-24 instrument (MP Biomedicals)

Refrigerated bench top centrifuge

Spectrophotometer

Wet chamber

Vacuum concentrator centrifuge

### Method

***1 Sample lysis***

- Add 200±10 mg glass beads and 200 µL lysis buffer to 30 mg cells (wet weight, equaling 10 OD _600_ units of yeast cells)
- Vibrate the cell suspension in a Fast Prep-24 instrument (4°C, settings: 6.5 Ms ^-1^, 20 sec). Repeat this step 2 times with a 5 min interval, keep samples on ice between intervals
- Centrifuge at 16,000 × *g* for 5 min
- Transfer the supernatant to a new reaction tube
- Centrifuge at 16,000 × *g* for 5 min
- Transfer the supernatant to a new reaction tube

***2 Protein quantification***

- Use BCA assay to determine the protein concentration according to the manufacturer’s instructions

***3 Sample processing***

**3.1 Protein separation by SDS-PAGE**

- Cast separating (15% acrylamid/bisacrylamide) and stacking gels (4%) according to Laemmli (Laemmli, UK. Cleavage of structural proteins during the assembly of the head of bacteriophage T4. Nature. 1970, **227**, 680–685)
- Add SDS-PAGE loading buffer to a lysate aliquot containing 50 µg protein and incubate at 95°C for 5 min to denature proteins
- Perform electrophoresis for 20 min at 100 V to allow the proteins to enter the separating phase
- Excise the sample from the gel in a single slice

**3.2 Excision and *in-gel* digestion of protein bands**

- Cut the gel slice into small pieces (1 mm) and place into a new reaction tube
- Add 100 µL (or enough to cover) 25 mM ABC/50% (v/v) ACN and vortex for 10 min
- Centrifuge at 16,000 × *g* for 30 sec and remove the supernatant using a gel-loading micropipette tip. Repeat this step 2 or 3 times
- Evaporate the solvents in a vacuum concentrator centrifuge (approximately 20 min)
- Add 50 µL (or enough to cover) 10 mM DTT in 25 mM ABC to the dried gel pieces
- Vortex and centrifuge at 16,000 × *g* for 30 sec
- Incubate with the reductive solution at 56°C for 1 h
- Remove the supernatant and add 50 µL (or enough to cover) 50 mM IAA to the gel pieces. Vortex and centrifuge at 16,000 × *g* for 30 sec
- Incubate with the alkylating solution in the dark for 30 min at room temperature, with occasional vortexing. Centrifuge at 16,000 × *g* for 30 sec
- Remove the supernatant. Add ~100 µL 25 mM ABC to the gel pieces. Vortex for 5 min and centrifuge at 16,000 × *g* for 30 sec
- Remove the supernatant and add ~100 µL (or enough to cover) 25 mM ABC/50% (v/v) ACN to dehydrate the gel pieces. Vortex for 5 min and centrifuge at 16,000 × *g* for 30 sec. Repeat this step
- Evaporate the solvents from the gel pieces in a vacuum concentrator centrifuge (approximately 20 min)
- Add 10 µL of trypsin (10 ng/µL) to the dried gel pieces and incubate for a few min to allow rehydration
- Add 25 µL 25 mM ABC (or sufficient volume to cover the gel pieces), vortex for 5 min, centrifuge at 16,000 × *g* for 30 sec and incubate at 37°C overnight in a wet chamber
- Centrifuge at 16,000 × *g* for 30 sec. Add 10 µL water, vortex for 10 min and centrifuge at 16,000 × *g* for 30 sec
- Transfer the tryptic peptides (aqueous extraction) into a new reaction tube
- Add 30 µL (or enough to cover) of 50% (v/v) ACN/5% (v/v) FA to the gel pieces, vortex for 10 min and centrifuge at 16,000 × *g* for 30 sec. Combine the supernatants of this and the previous step. Repeat this step once more
- Add 10 µL 100% (v/v) ACN to the gel pieces, vortex for 5 min and centrifuge at 16,000 × *g* for 30 sec. Combine with previous extractions
- Centrifuge the tryptic peptide mix at 16,000 × *g* for 30 sec and evaporate solvents in a vacuum concentrator centrifuge (approximately 2 h)
- Re-suspend the peptides in 50 µL 5% ACN/0.1% FA to obtain a final concentration of 1 µg/µL
- Aliquot the flow-through and store tryptic peptides at -80°C

**Compared to the original protocol the following changes were made:**

- Cell lysis and protein extraction was performed in 50 mM ABC
- The lysis of yeast cells was performed on a Fast Prep instrument

## Protocol 3: Filter-aided sample preparation (FASP)

(Adapted from Wisniewski, JR, Zougman, A, Nagaraj, N, Mann, M. Universal sample preparation method for proteome analysis. Nat. Methods 2009, **6**: 359–363)

### Materials

***Solutions and reagents***

Lysis buffer: SDT buffer (4% SDS, 100 mM Tris/HCl pH 7.6, 0.1 M Dithiothreitol (DTT, Melford Cat No. MB1015)

UA: 8 M urea in 0.1 M Tris/HCl pH 8.5

UB: 8 M urea in 0.1 M Tris/HCl pH 8

IAA: 0.05 M Iodoacetamide (Sigma Cat. No. I1149-5G) in UA

Lys-C: Sequencing grade Endoproteinase Lys-C (Stock 0.1 µg/µL; Promega Cat. No. V1071) in UB

Sequencing Grade Modified Trypsin: Stock 0.2 µg/µL (Promega Cat. No. V5111)

ABC: 0.05 M Ammonium bicarbonate (Fluka Cat. No. 40867-50G-F) in water

0.5 M NaCl

TFA: Trifluoroacetic acid (Sigma Cat. No. T6508)

FA: Formic acid

ACN: Acetonitrile

BCA Protein assay kit (Pierce Cat. No. 23225)

***Equipment***

Acid-washed glass beads (Sigma Cat. No. G8772-500G)

Fast Prep-24 instrument (MP Biomedicals)

Amicon Ultra-0.5 Centrifugal Filter Unit with Ultracel-3 membrane (Millipore Cat. No. UFC500396)

Protein LoBind tubes (Eppendorf Cat. No. 0030 108.094)

Empore SPE Cartridges C18 (standard density), bed I.D. 7 mm, volume 3 mL (Sigma Cat. No. 66872-U)

Ultrasonic tank (Langford Electronics Limited)

Refrigerated bench top centrifuge

Thermomixer (Eppendorf)

Spectrophotometer

Wet chamber

Vacuum concentrator centrifuge

### Method

***1 Sample lysis***

- Add 200±10 mg glass beads and 200 µl lysis buffer to 30 mg cells (wet weight, equaling 10 OD _600_ units of yeast cells)
- Vibrate the cell suspension in a Fast Prep-24 instrument (4°C, settings: 6.5 Ms ^-1^, 20 sec). Repeat this step 2 times with a 5 min interval, keep samples on ice between intervals
- Centrifuge at 16,000 × *g* for 1 min to sediment the cell debris
- Incubate for 3–5 min at 95°C
- Centrifuge as above and transfer the supernatant to a new reaction tube
- Sonicate for 5 min in an ultrasonic tank to reduce viscosity

***2 Protein quantification***

- Use BCA assay to determine the protein concentration according to the manufacturer’s instructions

***3 Sample processing***

**3.1 On-filter digestion**

- Apply 30 µL protein sample to the filter unit and add 200 µL UA
- Centrifuge at 14,000 × *g* for 40 min
- Apply 200 µL UA to the filter unit and centrifuge at 14,000 × *g* for 40 min
- Incubate for 5 min in the dark
- Centrifuge 14,000 × *g* for 30 min
- Apply 100 µL UB to the filter unit and centrifuge 14,000 × *g* for 40 min. Repeat this step two more times
- Apply 40 µL UB with Lys-C (enzyme to protein ratio 1:50) to the filter unit and mix at 600 rpm for 1 min at RT
- Incubate the units in wet chamber at 37°C overnight
- Transfer the filter unit to a new collection tube
- Apply 120 µL ABC with trypsin (enzyme to protein ratio 1:100) to the filter unit and mix at 600 rpm for 1 min at RT
- Incubate the filter unit at RT for 4 hrs
- Centrifuge at 14,000 × *g* for 40 min
- Apply 50 µL 0.5 M NaCl to the filter unit and centrifuge at 14,000 × *g* for 20 min
- Add TFA to reach a final concentration of 0.5% and remove salts from the filtrate

**3.2 Desalting of peptides**

- Place a 3 ml MILI-SPE Extraction disk cartridge (C18-SD) in a 15 ml conical tube
- Add 1 ml TFA and centrifuge at 1,500 × *g* for 1 min
- Add 0.5 ml of 0.1% TFA, 70% ACN in water and centrifuge at 1,500 × *g* for 1 min
- Add 0.5 ml of 0.1% TFA in water and centrifuge at 1,500 × *g* for 1 min
- Load the filtrate and centrifuge at 150 × *g* for 3 min
- Add 0.5 ml of 0.1% TFA in water and centrifuge at 150 × *g* for 3 min
- Transfer the cartridge to a new tube, add 0.5 ml of 70% ACN in water and centrifuge at 150 × *g* for 3 min
- Collect the flow-through that contains the desalted peptides
- Remove solvents in vacuum concentrator centrifuge and re-suspend in 30 µL 5% ACN/0.1% FA
- Aliquot and store tryptic peptides at -80°C

**Compared to the original protocol the following changes were made:**

- Yeast cells lysis was perfomed using a Fast Prep-24 instrument
- Amicon Ultra-0.5 Centrifugal Filter Unit with Ultracel-3 membrane was used (Millipore, Cat. No 42407)

## Protocol 4: eFASP

(Adapted from Shevchenko, G, Musunuri, S, Wetterhall, M, Bergquist, J. Comparison of extraction methods for the comprehensive analysis of mouse brain proteome using shotgun-based mass spectrometry. J. Proteome Res. 2012, **11**, 2441–2451)

### Materials

***Solutions and reagents***

Lysis buffer: 1% SDS, 10 mM Tris/HCl pH 7.4, 0.15 M NaCl, 1 mM EDTA in PBS

TBP: tri-n-butylphosphate (Fluka Cat. No. 90820-100ML)

Acetone

Methanol

ACN: Acetonitrile

Digestion buffer: 1% n-octyl-beta- d-glucopyranoside (nOGP, Sigma Cat. No. O8001) in 50:50 ACN/8 M urea

Complete EDTA-free Protease inhibitor Cocktail tablets Roche Cat. No. 11873580001). Dissolve one tablet in 1 mL water, to achieve a 50 x stock solution

0.09 M Dithiothreitol (DTT, Melford Cat No. MB1015)

0.1 M Iodoacetamide (IAA, Sigma Cat. No. I1149-5G)

Sequencing Grade Modified Trypsin: Stock 0.2 µg/µL (Promega Cat. No. V5111)

ABC: 0.05 M Ammonium bicarbonate (Fluka Cat. No. 40867-50G-F) in water

UPLC/MS grade water

FA: Formic acid

Acetic acid

BCA Protein assay kit (Pierce Cat. No. 23225)

***Equipment***

Glass Dounce homogenizer (Thomas Scientific Cat. No. 3432N75)

Amicon Ultra-0.5 Centrifugal Filter Unit with Ultracel-3 membrane (Millipore Cat. No. UFC500396)

Protein LoBind tubes (Eppendorf Cat. No. 0030 108.094)

Refrigerated bench top centrifuge

Spectrophotometer

Vacuum concentrator centrifuge

### Method

***1 Sample lysis***

- Add 200 µL lysis buffer to 30 mg cells (wet weight, equaling 10 OD _600_ units of yeast cells)
- Lyse cells in a glass Dounce homogenizer for 1 min
- Add 4 µL protease inhibitor solution
- Incubate the sample at 4°C for 1 h with mild agitation
- Centrifuge for 30 min at 10,000 × *g*, 4°C
- Transfer supernatant to a new reaction tube

***2 Sample Processing***

**2.1 Delipidation and protein precipitation**

- Mix 90 µL cell extract with 1.26 mL ice-cold TBP/acetone/methanol mix (1:12:1)
- Incubate at 4°C for 90 min
- Centrifuge at 2,800 × *g* at 4°C for 15 min
- Re-suspend the pellet in 1 ml TBP
- Centrifuge at 16,000 × *g* at 4°C for 15 min
- Re-suspend the pellet in 1 ml acetone
- Centrifuge at 16,000 × *g* at 4°C for 15 min
- Re-suspend the pellet in 1 ml methanol
- Centrifuge at 16,000 × *g* at 4°C for 15 min
- Evaporate solvents by incubating the sample at room temperature (RT) for 15 min
- Re-suspend the pellet in 100 µL digestion buffer

**2.2 Protein quantification**

- Use BCA assay to determine the protein concentration according to the manufacturer’s instructions

**2.3 On-filter digestion**

- Add 36 µL of 45 mM DTT to 30 µg protein (in 90 µL) and incubate at 50°C for 15 min
- Incubate samples at RT for 15 min
- Add 36 µL of 100 mM IAA and incubate at room temperature for 15 min in dark
- Apply 250 µL 50% ACN to the filter unit and centrifuge at 14,000 × *g* for 15 min
- Apply 500 µL water to the filter unit and centrifuge at 14,000 × *g* for 20 min
- Apply the protein sample to the filter unit and centrifuge for 15 min at 14,000 × *g* to remove salts and detergents
- Add 100 µL 2% ACN in 50 mM ABC and centrifuge at 14,000 × *g* for 10 min
- Add 100 µL 50:50 ACN/50 mM ABC and 100 µL 50 mM ABC, centrifuge at 14,000 *g* for 10 min
- Add 100 µL 50 mM ABC containing trypsin to achieve an enzyme to protein ratio of 1:40
- Incubate at 37°C overnight
- Centrifuge at 14,000 × *g* for 20 min and transfer the filtrate to a new reaction tube
- Add 100 µL 50% ACN/1% acetic acid to the filter and centrifuge for 10 min at 14,000 × *g*
- Combine with the previous filtrate
- Evaporate the solvents using a vacuum concentrator centrifuge (approximately 3 hrs)
- Re-suspend the tryptic peptides in 5% ACN/0.1% FA to obtain a final concentration of 1 µg/µl
- Aliquot and store tryptic peptides at -80°C

**Compared to the original protocol the following changes were made:**

- 90 µl of cell extract was subjected to delipidation
- 30 µg of protein were used for tryptic digestion

## Protocol 5: RapiGest

(Waters (UK), RapiGest-including version of a protocol based on der Haar, T. *Optimized protein extraction for quantitative proteomics of yeasts*. PLoS One 2007, **2**: e1078)

### Materials

***Solutions and reagents***

Lysis buffer: 0.1 M NaOH, 0.05 M EDTA, 2% SDS, 2% β-mercaptoethanol

ABC: 0.05 M Ammonium bicarbonate (Fluka Cat. No. 40867-50G-F) in water

DTT: 0.05 M Dithiothreitol (Melford Cat No. MB1015)

IAA: 0.1 M Iodoacetamide (Sigma Cat. No. I1149-5G) in ABC

Sequencing Grade Modified Trypsin: Stock 0.2 µg/µL (Promega Cat. No. V5111)

RapiGest SF Surfactant: Stock 0.2% in ABC (Waters Cat. No. 186002123)

Acetonitrile (ACN)

Acetic acid

Trichloroacetic acid (TCA)

Acetone

water, UPLC/MS grade

BCA Protein assay kit (Pierce Cat. No. 23225)

TFA: Trifluoroacetic acid (Sigma Cat. No. T6508)

***Equipment***

Protein LoBind tubes (Eppendorf Cat. No. 0030 108.094)

Refrigerated bench top centrifuge

Ultrasonic tank (Langford Electronics Limited)

Thermomixer (Eppendorf)

Spectrophotometer

Wet chamber

Vacuum concentrator centrifuge

### Method

***1 Sample lysis***

- Add 200 µL lysis buffer per 30 mg cells (wet weight, equaling 10 OD _600_ units of yeast cells)
- Incubate for 10 min at 90°C
- Add 5 µL 4 M acetic acid and vortex for 30 sec
- Incubate for 10 min at 90°C
- Centrifuge at 16,000 × *g* for 5 min
- Transfer the supernatant to a new reaction tube

***2 Protein precipitation***

- Add 205 µL 20% TCA to reach a final concentration of 10%
- Incubate for 2–3 hrs at -80°C to favor protein precipitation
- Centrifuge at 20,000 × *g* for 30 min at 4°C
- Re-suspend the pellet in 1 ml 80% acetone, solubilize by incubating for 5 min in an ultrasonic tank. Repeat this step until suspension is homogenous
- Incubate at 4°C for 1 h
- Centrifuge at 20,000 × *g* for 30 min at 4°C
- Re-suspend the pellet in 1 ml 80% acetone by sonicating for 5 min. Repeat this step until suspension is homogenous (Optional: store at -80°C overnight)
- Centrifuge at 20,000 × *g* for 30 min, 4°C
- Dry the pellet at room temperature (RT) for 10–30 min

***3 Solubilisation and Protein quantification***

- Add 100 μL 0.2% RapiGest in 50 mM ABC to the pellet
- Incubate for 10 min at 40°C at 400 rpm in a thermoshaker (keep tubes open to let acetone evaporate)
- Sonicate to dissolve the pellet using the ultrasonic tank (5 × 90 sec pulse, 30 sec pause on ice). Repeat this step until precipitate is fully dissolved and solution appears clear
- (Optional: if proteins remain insoluble, add 50 µL 0.2% RapiGest in 50 mM ABC to the sample and repeat sonication)

***4 Protein quantification***

- Use BCA assay to determine the protein concentration according to the manufacturer’s instructions

***5 Sample processing***

- Transfer 50 μg protein to a new reaction tube, fill up with 50 mM ABC to reach a total volume of 22.5 μL
- Add 2.5 μL 50 mM DTT in 50 mM ABC to reach a final concentration of 4.5 mM. Incubate at 60°C for 30 min
- Add 2.8 μL 100 mM IAA in 50 mM ABC and 0.2 μL 50 mM ABC to reach a final concentration of 10 mM
- Incubate in the dark for 30 min at RT
- Add 6.25 μL trypsin (enzyme to protein ratio 1:40)
- incubate 2 h at 37°C
- Add 6.25 μL trypsin (enzyme to protein ratio 1:40)
- Incubate at 37°C overnight
- Add 10% TFA to reach a final concentration of 0.5%
- Incubate at 37°C for 1 h
- Centrifuge at 20,000 × *g* for 10 min at 4°C
- Transfer the supernatant containing the tryptic peptides to a new reaction tube without disturbing the pellet
- Add ACN to reach a final concentration of 5%
- Centrifuge at 20,000 × *g* for 3 min
- Transfer supernatant to a new reaction tube
- Centrifuge at 20,000 × *g* for 10 min
- Adjust the volume with UPLC/MS water to obtain a final protein concentration of 1 µg/µL
- Aliquot and store tryptic peptides at -80°C

**Compared to the original protocol the following changes were made:**

- Protein pellets were re-suspended by sonication using RapiGest as surfactant
- Protein precipitation was performed using 20% TCA
- Protein pellets were washed twice using 80% acetone
- Trypsin was added in two sequential steps to reach a final enzyme to protein ratio 1:20

## Protocol 6: RapidACN

(Bluemlein, K, Ralser, M. Monitoring protein expression in whole-cell extracts by targeted label- and standard-free LC-MS/MS. *Nat. Protoc*. 2011, **6**: 859–869)

### Materials

***Solutions and reagents***

ABC: 0.05 M Ammonium bicarbonate (Fluka Cat. No. 40867-50G-F) in water

DTT: 0.09 M Dithiothreitol (Melford Cat No. MB1015)

IAA: 0.1 M Iodoacetamide (Sigma Cat. No. I1149-5G) in ABC

TCEP: 0.1 M *tris*(2-carboxyethyl)phosphine (Sigma Cat. No. C4706-2G)

Sequencing Grade Modified Trypsin: Stock 0.2 µg/µL (Promega Cat. No. V5111)

UPLC/MS water

FA: Formic acid

ACN: Acetonitrile

BCA Protein assay kit (Pierce Cat. No. 23225)

***Equipment***

Acid-washed glass beads (Sigma G8772-500G)

Amicon Ultra-0.5 Centrifugal Filter Unit with Ultracel-3 membrane (Millipore Cat. No. UFC500396)

Protein LoBind tubes (Eppendorf Cat. No. 0030 108.094)

Fast Prep-24 instrument (MP Biomedicals)

Refrigerated bench top centrifuge

Spectrophotometer

Wet chamber

Vacuum concentrator centrifuge

### Method

***1 Sample lysis***

- Add 200±10 mg beads to 30 mg cells (wet weight, equaling 10 OD _600_ units of yeast cells)
- Add 200 µL 50 mM ABC
- Vibrate the cell suspension in a Fast Prep-24 instrument (4°C, settings: 6.5 Ms ^-1^, 20 sec). Repeat this step 2 times with a 5 min interval, keep samples on ice between cycles
- Centrifuge 16,000 × *g* for 5 min
- Transfer supernatant to a new reaction tube
- Centrifuge at 16,000 × *g* for 5 min
- Transfer the supernatant to a new reaction tube

***2 Protein quantification***

- Use BCA assay to determine the protein concentration according to the manufacturer’s instructions

***3 Sample processing***

- Dilute the protein samples with 50 mM ABC to reach a final concentration of 2 µg/µL
- Add 25 µL 100% ACN to 25 µL protein sample (corresponding to 50 µg total protein) (A white precipitate might appear, do not remove it)
- Vortex thoroughly
- Incubate at 37°C for 15 min
- Add 5 µL 45 mM DTT/20 mM TCEP solution
- Incubate at 37°C for 30 min
- Add 5 µL IAA and incubate in the dark at room temperature for 30 min
- Centrifuge at 16,000 × *g* for 1 min
- Add 5.5 µL DTT to quench any residual IAA
- Add 134.5 µL UPLC-grade water
- Vortex and centrifuge at 16,000 × *g* for 1 min
- Add 6.25 µL trypsin enzyme to protein ratio 1:40)
- Incubate at 37°C for 2 hrs
- Centrifuge at 16,000 × *g* for 1 min and add 6.25 µL trypsin enzyme to protein ratio 1:40)
- Incubate at 37°C overnight in a wet chamber
- Centrifuge at 16,000 × *g* for 1 min
- Dry the sample using a vacuum concentrator centrifuge (approximately 2 hrs)
- Re-suspend in 50 µL 5% ACN/0.1% formic acid
- Add 0.7 µL 10% FA, vortex and centrifuge at 16,000 × *g* for 1 min
- Incubate at room temperature for 5 min
- Recommended optional clean-up: Apply sample to a spin filter unit and centrifuge according to the manufacturers instructions
- Aliquot the flow-through and store tryptic peptides at -80°C

**Compared to the original protocol the following changes were made:**

- Trypsin was added at a ratio protein:enzyme ratio of 1:20
- Trypsin was added in two sequential steps
- After lyophilisation peptides were resuspended in 50 µl 5% ACN/0.1% FA instead of 150 µl
