# Supplementary material for: The beauty of being (label)-free: sample preparation methods for SWATH-MS and next-generation targeted proteomics
Source: F1000Res. 2014 Apr 7;2:272. Originally published 2013 Dec 13. [Version 2] doi: 10.12688/f1000research.2-272.v2 (PMC3983906; doi:10.12688/f1000research.2-272.v2)
Supplement: Peer review history

# Peer review: Referee response for version 2

**DOI:** 10.5256/f1000research.4122.r4388

**Published:** 2014-05-22

**Authors:** Nick Morrice

**Affiliations:** 1 The Beatson Institute for Cancer Research, Glasgow, UK

The authors have made all the modifications I suggested and I have no hesitation in approving the revised manuscript.

I have read this submission. I believe that I have an appropriate level of expertise to confirm that it is of an acceptable scientific standard.

# Peer review: Referee response for version 2

**DOI:** 10.5256/f1000research.4122.r4387

**Published:** 2014-05-12

**Authors:** Xianyin Lai

**Affiliations:** 1 Department of Cellular and Integrative Physiology, Indiana University, Indianapolis, IN, USA

The manuscript has been revised according to the reviewer's comments.

I have read this submission. I believe that I have an appropriate level of expertise to confirm that it is of an acceptable scientific standard.

# Peer review: Referee response for version 1

**DOI:** 10.5256/f1000research.3109.r3833

**Published:** 2014-03-04

**Authors:** Nick Morrice

**Affiliations:** 1 The Beatson Institute for Cancer Research, Glasgow, UK

This is a well written manuscript and provides an excellent reference for sample preparation ahead of any label free proteomics experiment. The protocols in the supplementary section are very detailed making them very easy for both novice and expert researchers to follow. The manuscript basically compares three different types of sample preparation and describes the positives and negative for each protocol if applied to label free quantification of proteins. This manuscript will be a very useful reference for anyone who wishes to perform label free quantification of proteins as the authors do highlight both the advantages and disadvantages of each approach.

The minor revisions I would like to see are in the description of creating a SWATH ion library using offline high pH RP-HPLC. The authors mention the addition of iRT peptides to each fraction, but do not describe why they are added. These retention time reference peptides are added so that any retention time drift from the ion library to the samples can be accounted for by the software used to analyse and quantify the SWATH data. I feel this should be described, especially for novices to the field. Secondly there is a slight complication in using the term eFASP in this manuscript. A very recent publication by Erde, J. *et al.* (2014) uses the term eFASP to describe a method that differs significantly from the one described here. I think this paper should be referred to in this manuscript just to highlight that the eFASP method used here was based on the method described by Shevchenko *et al.* (2012) and not to be confused with this more recent publication. Apart from these minor corrections, I have no reservations in approving this article.

I have read this submission. I believe that I have an appropriate level of expertise to confirm that it is of an acceptable scientific standard.

# Response

**Published:** 2014-04-07

**Authors:** Markus Ralser

**Affiliations:** Cambridge Systems Biology Centre (Department of Biochemistry), University of Cambridge, UK

We thanks for these suggestions, and for spotting out the nomenclature overlap with Erde *et al. *(2014). As the Erde *et al.* article was published ~three months after the first version of this article we decided to keep the nomenclature, but add a caveat for clarification on two instances in the paper. An explanation/rationale about the use of retention time normalisation standards is now included as well.

# Peer review: Referee response for version 1

**DOI:** 10.5256/f1000research.3109.r3900

**Published:** 2014-02-27

**Authors:** Xianyin Lai

**Affiliations:** 1 Department of Cellular and Integrative Physiology, Indiana University, Indianapolis, IN, USA

In their manuscript, Vowinckel *et al*. evaluated 6 sample preparation methods for label-free quantification, compared DDA and SWATH approaches of TripleTOF5600, and drew two major conclusions. The organization of the manuscript is clear, it is well-written, and the coverage is effective. The first conclusion is supported by their results. However, the second conclusion should be clarified that SWATH outperformed DDA in quantification when a TripleTOF5600 was applied. Without the clarification, it misleads scientists to believe that SWATH outperforms all DDA approaches carried out using other mass spectrometers. Figure 2C shows that DDA of QExactive detected about 40% more proteins than SWATH of TripleTOF5600. It is necessary to compare DDA of QExactive with SWATH of TripleTOF5600.

**Minor issues:**

- "Protein Discoverer" should be "Proteome Discoverer"

I have read this submission. I believe that I have an appropriate level of expertise to confirm that it is of an acceptable scientific standard.

# Response

**Published:** 2014-04-07

**Authors:** Markus Ralser

**Affiliations:** Cambridge Systems Biology Centre (Department of Biochemistry), University of Cambridge, UK

We thank the reviewer for the suggestion and clarify this now. Indeed,  the TripleTOF5600 and Qexactive data can only to be compared indirectly/correlative in this study, but not absolutely, as different chromatography, HPLC, and ionisation settings were used. Therefore, the study can not be used to compare the Qexactive and TripleTOF instruments. However, in relative terms the protocols perform equally on both platforms, see Figure 2d, inset.  As suggested, we have improved this part in manuscript and abstract, and included the Reviewer's comments.
